# Identifying Implementation Factors for the Development, Operation, and Sustainment of Ambulatory Care Pharmacy Programs: a Qualitative Study

**DOI:** 10.1007/s11606-023-08375-1

**Published:** 2023-08-24

**Authors:** Nabeel Qureshi, Michelle S. Keller

**Affiliations:** 1 RAND Corporation, Santa Monica, CA USA; 2 Pardee RAND Graduate School, Los Angeles, CA USA; 3 Division of General Internal, Department of Medicine, Cedars-Sinai Medical Center, Medicine-Health Services Research, Los Angeles, CA USA; 4 Department of Health Policy and Management, UCLA Fielding School of Public Health, Los Angeles, CA USA

**Keywords:** pharmacy, implementation science, outpatient

## Abstract

**Background:**

Pharmacist-led programs and clinics have been integrated into primary and specialty care clinics in a variety of ways, for example, to improve diabetes outcomes via patient education and counseling. However, factors important to the implementation of different outpatient pharmacy models have not been well elucidated.

**Objective:**

To identify provider- and health system–level drivers of implementation and sustainability of pharmacy-led programs in the outpatient setting.

**Design:**

Qualitative study of key informants using semi-structured interviews of individuals working in various roles throughout a large health system, including ambulatory clinical pharmacists, pharmacy managers, medical directors and physician leaders, and operations and quality managers.

**Participants:**

Key informants (*n*=19) with leadership roles in pharmacy programs and front-line experience providing integrated pharmacy care were selected purposively and with snowball sampling.

**Approach:**

We coded the interviews using a codebook derived from the 2022 Consolidated Framework for Implementation Research (CFIR), which details various internal and external factors important for implementation.

**Key Results:**

We identified the following themes related to implementing ambulatory care pharmacy programs: (1) pharmacy programs varied in their level of embeddedness in the outpatient clinic, (2) establishing pharmacy program required leadership advocacy and coordination among stakeholders, (3) continued operations required integrated workflows and demonstrated value to the health system and clinicians, and (4) established revenue streams or added indirect value and continued improvement of integration sustained programs over time.

**Conclusions:**

External policies and incentives such as new reimbursement codes and quality measurement programs that rely on pharmacy input play a significant role in shaping the design, implementation, and sustainability of health system outpatient pharmacy programs. Ensuring that quality metrics used in value-based contracts or programs demonstrate pharmacy benefits will be critical to supporting and growing pharmacy programs.

**Supplementary Information:**

The online version contains supplementary material available at 10.1007/s11606-023-08375-1.

## INTRODUCTION

Pharmacists’ contributions to improving quality of care have been increasingly recognized in the ambulatory care setting. As medication regimens have increased for chronic diseases^1^ and primary care providers are increasingly burdened with additional tasks,^2^ there has been a shift to supporting primary care activities through other providers.^3^ Pharmacists have been shown to play important roles in reviewing whether prescribed medications are appropriate, improving medication adherence,^4^ and counseling patients on lifestyle interventions. Integration of pharmacists into outpatient care is an evidence-based practice (EBP) that is increasingly being adopted.^5^ Two recent systematic reviews found that pharmacist services in the ambulatory clinic setting resulted in improved blood pressure, glycosylated hemoglobin, and cholesterol measures.^3,4^ With the rise of value-based care innovations, which often measure quality through clinical measures modifiable via pharmacist interventions,^6^ integrating pharmacy services in primary and specialty care may become an increasingly valuable strategy to improve quality of care. Moreover, as the population in the USA and other countries continues to age and face increasing issues such as polypharmacy,^5,7,8^ demand for pharmacists will likely increase.

Despite evidence demonstrating the clinical effectiveness of embedding pharmacists in primary and outpatient specialty care practices, operational leaders face a variety of barriers when integrating pharmacists into clinics, particularly in the USA. These challenges may include space for pharmacists to work; staff support for pharmacists; changes in workflows; buy-in from stakeholders, including managers, patients, physicians, medical assistants, and nurses; specialty certifications; and scope of practice.^9–11^ Critically, as pharmacists do not have “provider status” in the USA under Medicare Part B, they are unable to receive reimbursement for services within their scope of practice. Although the majority of states already recognize pharmacists as providers, there is extremely varied state-by-state implementation of scope of practice or reimbursement practices for which services pharmacists could receive payment.^12^ This has resulted in the need to create a variety of different payment and reimbursement models to sustain pharmacy services. Scope of practice is another major barrier: not all states allow initiation of medications in the outpatient setting, some only allow modification of medication regimens, and others only allow initiation in inpatient settings only.^13^

Given these existing challenges, we sought to understand how a large health system designed, implemented, and worked to sustain a variety of ambulatory pharmacy programs and models. We used the 2022 Consolidated Framework for Implementation Research (CFIR), a framework that combines implementation-related constructs that influence implementation and implementation effectiveness,^14^ to understand barriers and facilitators to pharmacy integration across a range of programs available at the health system. Our objective was, first, to describe the types of pharmacy programs that had been implemented within one large health system with multiple hospitals and outpatient clinics, and second, to identify which implementation factors drove the implementation and sustainability of ambulatory pharmacy programs and models, with the goal that these research findings could be used to design and implement new pharmacy programs.

## METHODS

### Setting, Participants, and Study Design

We selected a qualitative approach to provide insights into various factors within and outside of organizations that drive the implementation and sustainability of outpatient pharmacy programs to capture the breadth of factors and experiences that supported these programs. The study was conducted in a large outpatient health system, Cedars-Sinai Medical Care Foundation (CSMCF), associated with a large academic medical center, Cedars-Sinai Medical Center (CSMC), located in Los Angeles, California. CSMCF is composed of several private medical groups, including the Cedars-Sinai Medical Group, a managed care medical group with more than 100 primary care physicians, and the Cedars-Sinai Health Associates, an independent practice association with more than 400 private physicians.

**Figure 1:**
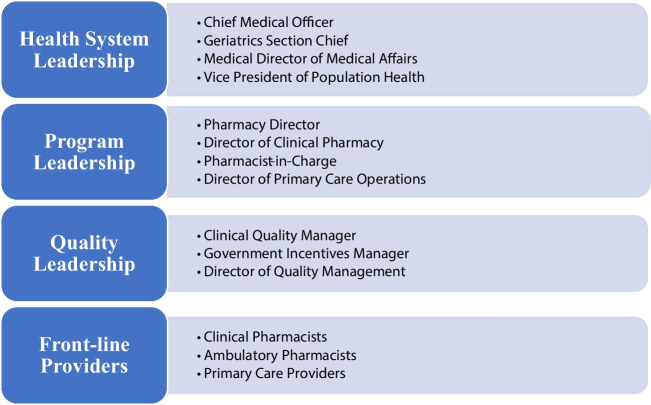
Overview of interview sample by category.

We conducted in-depth, semi-structured interviews with a purposively selected group of stakeholders in CSMCF whom we hypothesized would be involved with the implementation of pharmacy programs, followed by individuals identified through snowball sampling of the selected stakeholders. We intentionally sought out participants involved at various levels of the organization, including front-line providers such as clinical pharmacists and primary care physicians, pharmacy managers, operational managers, and clinical and operational leaders, grouped by system, leadership, program leadership, quality leadership, and front-line staff (Fig. 1). Given the roles of individuals identified, stakeholders had experience working across multiple pharmacy programs and provided insights to each program during the interview. We assessed data saturation when there were no new themes emerging.

We asked for verbal consent to participate and record from all interview participants. Individuals were asked if they would be open to follow up discussions after each interview. All interviews were conducted via Zoom, and were audio recorded, transcribed by a professional transcription, and de-identified. Two investigators with experience with qualitative research (MSK and NQ) who work as researchers in the health system conducted all the interviews. The qualitative study was reviewed and approved by the Cedars-Sinai Institutional Review Board.

### Interview Guide

The semi-structured interview guide was informed by the CFIR framework^14^ available in the Appendix. We used the topic guide to generate questions and tailor interview protocols for each stakeholder about the description of the existing pharmacy programs, implementation of existing and past pharmacy programs, changes to care delivery associated with implementing the programs, factors that facilitated or served as barriers in the implementation or expansion, adaptions to existing programs, and sustainability or expansion of pharmacy services.

### Qualitative Analysis

We created an a priori codebook using the domains and constructs of CFIR^14^ and used the codebook to code the transcripts in Dedoose (SocioCultural Research Consultants, version 9.0.17). We inductively identified themes related to facilitators and barriers for integrating pharmacy programs into outpatient care using a practical thematic analysis approach.^15^ We mapped themes to coded CFIR domains and constructs from the interview transcripts to identify the factors that most support or hinder implementation. We met regularly to discuss emerging themes based on interviews and coding and compared themes across pharmacy programs and stakeholder type. For example, we compared different pharmacy clinic structures (embedded vs. non-embedded) and how these structures led to differences in workflows, adoption, expansion, and sustainability. We identified factors from the CFIR framework that were regularly mentioned by stakeholders and mapped those to the emergent themes. Reporting of the results of the study follow the Standards for Reporting Qualitative Research (SRQR) Guidelines.^16^

## RESULTS

### Overall Sample

We conducted 17 individual and two group interviews (with two participants each) for a total of 19 interviews with 20 administrators and clinical staff working across the health system, including clinical pharmacists (*n*=6, including one pharmacist who was interviewed once alone and once in a group interview to collect additional information), pharmacy managers (*n*=4), medical directors and physician leaders (*n*=5), and operations and quality managers (*n*=4). Individuals varied in their level of experience at the organization, from 3 to 24 years in the health system. See Table 1 for a description of participants.

**Table 1:** Interview Sample Description

Interview ID	Role	Specialty (if applicable)
Participant 1	Clinical pharmacist	Pharmacist
Participant 2	Clinical pharmacist; senior manager of clinical pharmacy and nutrition services	Pharmacist
Participant 3	Chair department of primary and acute care	Internal medicine
Participant 4	Clinical pharmacist	Pharmacist
Participant 5a	Clinical pharmacist	Pharmacist
Participant 5b	Ambulatory pharmacy manager; pharmacist-in-charge	Pharmacist
Participant 6	Ambulatory pharmacist	Pharmacist
Participant 7	Medical director of medical affairs	Internal medicine
Participant 8	Clinical pharmacist	Pharmacist
Participant 9a	Pharmacy director	Pharmacist
Participant 9b	Clinical pharmacist	Pharmacist
Participant 10	Manager, government incentives	NA
Participant 11	Manager, quality	NA
Participant 12	Director, primary care operations	NA
Participant 13	Director, quality management	NA
Participant 14	Section chief, geriatrics	Geriatrics
Participant 15	Chief medical officer	Internal medicine
Participant 16	Internal medicine physician	Internal medicine
Participant 17	Internal medicine physician	Internal medicine
Participant 18	Executive director, pharmacy and nutrition services	Pharmacist
Participant 19	Vice president, population health	NA

### Pharmacy Programs Varied in Their Level of Embeddedness in the Clinic

In interviews with key informants, we identified two types of pharmacy programs that existed in the outpatient setting. These programs emerged as having their own unique structures and a distinct set of factors related to the successful implementation of these programs. Pharmacy programs in the outpatient setting were either (1) pharmacy programs embedded and integrated in specific clinics (i.e., primary care or specialty) and their workflows (herein called “embedded pharmacy programs”) or (2) pharmacy programs that were outside of a specific clinic and were available to providers who work in the Independent Physician Association (IPA) and Health Maintenance Organization (HMO) (herein called “non-embedded pharmacy programs”). Multiple programs existed within each group. For example, embedded pharmacy programs included pharmacists embedded in primary care clinics who addressed conditions such as diabetes and hypertension and pharmacists embedded in neurology and rheumatology clinics. Non-embedded pharmacy programs included a benzodiazepine-tapering clinic, a general refill center, a travel medicine clinic, and a pain management clinic.

Embedded pharmacy programs were defined as being located within a single medical clinic or accessible to a specific specialty group, such as a set of primary care clinics, and whose pharmacists worked in tandem with the clinicians in the clinic only. These programs were implemented within clinics due to leadership identification of their need (domain: *inner setting –* construct: *relative priority*), such as a system-wide primary care redesign effort that embedded general pharmacists into primary care clinics aimed at targeting diabetes medication optimization and specialty pharmacists embedded into rheumatology offices where medication support and assistance (e.g., medication selection and prior authorization management) are a large component of patient care. These embedded pharmacy programs were developed based on leadership decisions to improve care delivery overall, with a focus on targeting areas with the greatest medication support need (domain: *outer setting –* construct: *culture: recipient-centeredness*). For example, the desire to improve medication adherence HEDIS measures in primary care was one of the motivations to integrate pharmacists in primary care clinics (domain: *outer setting* – construct: *performance measurement pressure*).

In contrast, non-embedded pharmacy programs were available to all clinicians in the outpatient setting. Pharmacists were not embedded in a specific clinic and were generally pharmacists with specialty training (i.e., experience treating migraines, prescribing controlled substances) and dealt with conditions that touched many different types of providers. Development and financing models for non-embedded programs varied considerably and were most often driven by a champion in the health system identifying an unmet patient need. For example, the migraine clinic was developed by a primary care physician leader due to access issues in neurology and the availability of a pharmacist with training in treating migraine. The financing of the pharmacist for this program was done through improved patient quality of care, but other programs such as the travel medicine program used increased reimbursement from services to finance the program.

### Establishing Pharmacy Programs Required Leadership Advocacy and Coordination Among Stakeholders

The key implementation facilitators associated with the establishing an outpatient pharmacy program were leadership or champion advocacy for the pharmacy programs (domain: *process – assessing needs and engaging: innovation deliverers*, construct: *innovation recipients*), consistency of the program to the organization’s mission or priorities (domain: *inner setting –* construct: *relative priority*), coordination between the medical and pharmacy leadership (domain: *inner setting –* construct: *relational connections*), and the perceived ability to either monetize programs or provide additional value to patients (construct: *innovation relative advantage*)*.* We have summarized key implementation factors in Table 2.

**Table 2:** Key Factors Impacting Implementation and Relevant CFIR Constructs

Themes	Factors	CFIR construct
Establishing pharmacy programs required leadership advocacy and coordination among stakeholders	Leadership or champion advocacy for programs	Process – assessing needs and engaging: innovation deliverers, innovation recipients
	Consistency of programs with organization’s priorities	Inner setting – relative priority
	Level of coordination between medical and pharmacy leadership	Inner setting – relational connections
	Perceived ability to monetize programs or provide additional value	Innovation relative advantage
Continued operation required integrated workflows and demonstrated value to clinicians	Ability to integrate into clinical workflow	Innovation adaptability
	Demonstration of value to referring providers	Innovation design, outer setting – policies and laws, outer setting – external pressure: performance measurement pressure
	Level of embeddedness into clinical care	Inner setting – compatibility
Identifying revenue streams and continued integration and improvement sustained programs over time	Ability to finance programs (i.e., via reimbursement or through government/payor programs)	Innovation cost
	Ability to tie pharmacy activities to outcomes	Process – reflecting and evaluating, outer setting – policies and laws, outer setting – external pressure: performance measurement pressure
	Continued support from leadership or champion	Process – innovation deliverers and recipients
	Integration of pharmacy activities into routine care	Innovation adaptability
		Process – doing

Leadership or champion support was critical for the establishment of outpatient pharmacy programs. Given the lack of pharmacy presence in most clinics historically, leadership support was necessary to align various stakeholder goals (i.e., improve access to services, reduce time burden on physicians, improve quality metrics) to implement change. As this organization underwent a large-scale primary care transformation, health system leaders reported that their support in embedding pharmacists, which aligned with other priorities within the organization, improved quality of care for patients by addressing issues with medication adherence.

The evolution of ambulatory pharmacy from the beginning, when we had one pharmacist to where we are now, where I have a team of eighty people… It’s amazing. The origin really was chronic disease management… And so it started with diabetes. Primary care doctors are managing patients with chronic illness, and it’s mostly medication management. It’s a lot of titrating meds figuring out just the right dosing and trying the step therapies and that kind of thing. And so it was really [leader in organization], who was our founding physician, who partnered up with [Chief Pharmacy Officer], and they came up with the idea of let’s put an ambulatory pharmacist in at the medical group, and then that’s just sort of evolved from there… So the concept of pharmacists as physician extenders really took off in our group. – Health System Leader

In addition to leadership or champion support, close coordination between those leaders and pharmacy leadership was necessary to ensure programs were established. Pharmacy leadership had a unique understanding for how pharmacists practice and operate, and they provided insights into where pharmacists can be most effective and the necessary training for pharmacists to effectively embed in clinics. The pharmacy leadership also discussed how they could champion pharmacist programs to clinical groups which could benefit from a pharmacy program.

Key informants also discussed the need to monetize programs or provide additional value to patients from the pharmacy programs to establish these programs. Pharmacists and system leadership both reported a key barrier to establishing pharmacy programs being the inability of pharmacist to bill for their services under Medicare. As such, programs needed separate funding streams or ability to monetize pharmacy services to be financially viable to the health system. Several embedded pharmacy programs used a model of co-visits, where patients meet primarily with the pharmacist to discuss medications and treatment options and very briefly with the physician. The physician is “double booked” in that they have another patient whom they are seeing during this visit, but are able to check in with the pharmacist/patient and answer questions. These visits could be billed by the physician and would allow the physician to shift the management of low acuity patients with certain medications or treatments to the pharmacist, thereby freeing up the physician’s time to see additional, higher acuity patients. The co-visit model has also allowed primary care physicians to grow their panels over time and increase their relative value units (RVUs) while ensuring that patients with chronic diseases are well managed.

So, what we call it is a ‘co-visit.’ And that means that the patient will get scheduled with the pharmacist. But in addition, they will be scheduled as an overbook on the physician’s schedule. So, again the physician will have to spend an extra five minutes with this patient that otherwise they would not be seen with the pharmacist and be able to bill for that. – Clinical Pharmacist

In contrast, the majority of non-embedded pharmacy programs did not utilize co-visits as they were not co-located with medical providers. In most cases, these programs filled a need for patients that could not be met through the medical system. For example, when health system leaders identified the high use of long-term benzodiazepine among older adults in the health system, pharmacists and operational leaders designed and implemented a benzodiazepine-tapering clinic with a pharmacist trained to prescribe these medications. As benzodiazepines can take weeks to months to taper down and may require substantial patient support, pharmacists filled a critical need in providing this service. Physicians referring patients to this clinic signed a collaborative practice agreement, which allowed the pharmacists to participate in collaborative drug therapy management.

### Continued Operation Required Integrated Workflows and Demonstrated Value to System and Clinicians

The key implementation factors associated with the ongoing function of the outpatient pharmacy programs were ability to integrate pharmacy care into regular clinical workflows (*innovation adaptability*), demonstration of the value of pharmacy programs to referring providers (*innovation design*, *outer setting – policies and laws, outer setting – performance measurement pressure*), and alignment of the level of embeddedness of the pharmacy program with referring providers’ expectations (*inner setting* – *compatibility*).

Pharmacy programs used various methods to integrate into regular clinical workflows to cement themselves into the organization. Embedded pharmacists worked to make themselves known to clinicians in their clinic, reaching out as new clinicians join a clinic and regularly interfacing with clinicians to remind them of their services. Upon onboarding, clinicians shadow pharmacists to understand how the pharmacy programs work. Pharmacists also shared their capabilities and worked with clinicians to set up informal workflows to refer patients and set up norms around documentation and patient sharing. One pharmacist working with patients in a non-embedded opioid maintenance and benzodiazepine-tapering clinic noted that they try to promote and engage physicians to improve hand off processes.

What I’ve tried to do is when I noticed that one of the providers did a really great job setting up the patient well, and I can clearly review the referral, see the notes and the documentation, they signed the agreement, they checked a urine screen, and everything was appropriate... Yeah, I highlight what’s worked really well, and just be able to share for those physicians, outreach to them directly and say, thank you so much for doing that, it was really helpful. – Clinical Pharmacist

Clinical pharmacists also disclosed the variety of approaches they used to demonstrate their value. Chief among their approaches is noting the time saved by clinicians working with pharmacist for patients with many medications or complicated medication regiments. These efforts open up physician time to see additional patients and bill for additional patient time. Pharmacists also noted their knowledge of a wider range of medications, allowing for more tailored treatments for patients, especially for conditions that have a range of drugs. Pharmacists in specialty clinics noted their ability to stay up-to-date on new medications and having more time to discuss and update treatment for patients than clinicians.

In addition to direct forms of financing for pharmacist programs, pharmacy and system leadership discussed the role of pharmacists in supporting the health system in improving medication adherence measures related to the Medicare Advantage Star Rating system and other value-based programs, such as the Medicare Primary Care First Model. These efforts included patient outreach and counseling to increase medication adherence rates and de-prescribing prescriptions when they were no longer needed. Their efforts were used to increase reimbursement across the board through improved Star ratings and greater reimbursement for Medicare Advantage patients.

...when we sort of were looking at how to improve our Star measures a number of years ago, I would say I really championed pharmacy taking the lead in solving that problem. And they really have sort of been working closely with our health plans… in really fixing our Star measures by sort of aligning very closely and monitoring those measures and that database very closely every month. – Geriatrician

Pharmacist also noted that since they are not the source of their own patients, they need to align their work with the expectation of referring clinicians. Clinicians have a range of approaches and styles they use for managing patients, and varying expectations for the role of pharmacy. One pharmacist working with several neurologists noted the contrast between different providers and how each had different views of the role of the pharmacist overall and how the pharmacist should treat their patients.

So, it kind of comes back to, probably the complexity of the patient, and also, preference of the Neurologists. So, we have one of our [Clinician #1], she prefers all the patients to be seen as co-visits. Then we have one of the [Clinician #2] who prefers a patient who are very complex and require a lot of time to be spent with them, to spend that time as solo visits rather than co-visits, because that cannot really be a double booked on their schedule. So those are some of the things that I need to consider before requesting appointments. – Clinical Pharmacist

While most of the work pharmacists noted was needed to build buy-in with clinicians, pharmacists also noted instances where developing patient buy-in was necessary. Pharmacists embedded in clinics who perform co-visits with clinicians noted some patient reluctance to seeing a pharmacist rather than a physician for their medical needs. Strong relationships with the clinician, who can advocate on behalf of the pharmacist, allowed for the pharmacist to build support with the patient, then allowing the pharmacist to build their own relationship with the patient.

I think patients tend to be really grateful because we spend a lot of time explaining things. And things that we hope that they’ve been told before. But then a lot of people are like, ‘No one’s ever taken the time to explain what diabetes is and how much, and what medications do.’ – Ambulatory Pharmacist

### Established Revenue Streams or Added Indirect Value and Continued Improvement of Integration Sustained Programs over Time

Finally, the key implementation factors associated with sustaining pharmacy programs were the ability to continue financing the pharmacy program (construct: *innovation costs*), ability to tie outcomes to pharmacy activities (domain: *process –* construct: *reflecting and evaluating*, domain: *outer setting –* construct: *external pressure: performance measurement pressure*), continued support from leadership or champion (domain: *process –* construct: *innovation deliverers and recipients*), and ongoing integration of pharmacy activities with routine care (domain: *innovation adaptability*, construct: *process - doing*).

Given the inability of pharmacists to bill their services, the ability to provide some other value, either monetary (i.e., greater patient volume for physicians through co-visits, or improved quality related to value-based contracts and Medicare Advantage Star Ratings) or non-monetary (i.e., improved patient outcomes, or provider satisfaction), proved critical for sustainment. Clinician experience and buy-in to pharmacy programs positively relate to sustainment, as clinicians came to rely on and benefit from pharmacist activities.

…the clinical pharmacists are great. There’s so much they help us with. You know, even when some of the new rules about trying to give out some of these oral agents for COVID, they jumped right in to help. It made it much easier for the physicians then to be able to do this. So, there’s probably a myriad of ways the pharmacist, their value is just not even, has no idea being measured correctly. They are very valuable. – Medical Director

While some models like the co-visit model could be tied directly to pharmacist work, other improved outcomes such as improvement in quality of care were viewed as harder to tie to pharmacist activities. One pharmacist leader noted the difficulty in tying direct patient care pharmacy activities to outcomes.

So, it’s more kind of like we have to prove our worth by the quality we provide. So, for the migraine program, we implemented this migraine cocktail treatment through the pharmacist in the office. So, potentially saying, ‘Well, they would’ve gone to the emergency room if we didn’t treat them in the office.’ Potentially money is saved there. So, we always have to show that we’re saving something, somewhere. – Senior Manager of Clinical Pharmacy

In contrast, a lack of ongoing support was seen as a barrier to sustaining pharmacy programs over time, particularly those that are not embedded within a single clinic. For example, the loss of a physician champion could lead to a non-embedded clinic being closed or repurposed for another program. Finally, pharmacy programs needed to continue being integrated into regular clinical workflows to ensure these programs would persist. Medical providers noted that pharmacy programs that were easily accessible, either through simple referrals or co-location, and those that were beneficial to their patients, were of the most use to medical providers.

[The pharmacists] just join and it’s there and it’s a well-oiled machine. And it’s just part of the workflow that they’re told about from day one. And our pharmacists are extremely professional, and things work well. So, I don’t see any problem with accepting their input or their expertise. They’ve always been extremely responsive. - Physician

## DISCUSSION

Pharmacy programs have been shown to be effective in improving patient outcomes in the outpatient setting. However, operational constraints and policy choices around provider status have limited the ability to integrate pharmacy into clinical care. We sought to understand how pharmacy programs are implemented given the existing barriers to their implementation. In this qualitative study of key informants in a large health system with a variety of outpatient pharmacy programs, we identified a range of factors that supported the implementation of programs. Using the CFIR model, we identified two types of pharmacy programs—those embedded in clinics and those non-embedded pharmacy programs available to the larger provider base at the health system.

Pharmacy programs required leadership engagement early in the process, often advocating for programs that aligned with organizational priorities, and sustained advocacy as the program matured. Continued success via coordination between medical and pharmacy leadership was used to build the program and increase buy-in among both medical and pharmacy providers. Finally, programs required a mechanism for early and continued financing, which was limited by the inability to bill for pharmacy services. In lieu of direct mechanisms for financing, indirect forms such as improved quality scores associated with pay-for-performance programs or increasing access to primary care physicians by offloading certain activities (e.g., diabetes medication management) provided the type of benefit that was associated with ongoing support. As more value-based contracts emerge and medication-related measures are added to these contracts, there will be more tangible opportunities for pharmacist activities to be financed and supported. Once in place, pharmacy programs needed to integrate into clinical workflows. Pharmacists worked with physicians to establish norms around referral processes and clinical support, and physicians were acclimated to pharmacy services and workflows through shadowing. Finally, pharmacy programs need to be financed and show ongoing value and support from leadership or champions. As health system leadership considers integrating pharmacy programs into outpatient care, attention should be paid to the potential value of the program, with a clearly established strategy to finance and sustain the program, and integration with workflows to minimize perceived costs to providers.

This study has several limitations. First, this study focuses on one health system in an urban setting and results may not generalize broadly. However, given the health system’s investment in pharmacy programs, this study does examine a range of programs that strengthen the generalizability of the results. Second, our study focused on successfully implemented outpatient pharmacy programs. Finally, while the CFIR model is useful to identify factors that support or hinder implementation, they do not indicate whether factors are necessary or sufficient for successful implementation. However, understanding of implementation factors does help develop pharmacy programs that are more likely to succeed in practice.

Future research is needed to identify policies and practices that lead to greater penetration of pharmacy practice and quantify their impact on practice and outcomes in outpatient care delivery. Additionally, our study identifies factors related to success from the provider and health system leadership perspectives. Future inquiry should evaluate patient perceptions of embedding clinical pharmacists into outpatient care delivery.

### Supplementary Information

Below is the link to the electronic supplementary material.

Supplementary file1 (DOCX 25 KB)

## Data Availability

The datasets generated during and analyzed during the current study are not publicly available due the data being easily identifiable.
