# Supplementary material for: Identifying Implementation Factors for the Development, Operation, and Sustainment of Ambulatory Care Pharmacy Programs: a Qualitative Study
Source: J Gen Intern Med. 2023 Aug 24;38(15):3381–8. doi: 10.1007/s11606-023-08375-1 (PMC10682305; doi:10.1007/s11606-023-08375-1)
Supplement: Supplementary file 1 — Supplementary file1 (DOCX 25 KB) [file 11606_2023_8375_MOESM1_ESM.docx]

**Appendix.** Topic guide and associated concepts from the updated Consolidated Framework for Implementation Research

***Characteristics of Individuals – Knowledge and Beliefs about the Intervention***

***Characteristics of Individuals – Self-efficacy***

- Knowledge about existing ambulatory pharmacy program(s)
- Role in ambulatory pharmacy program(s)
  - What sold the individual on need for ambulatory pharmacy program(s) (motivations)

***Innovation Design, Complexity, Evidence Base, Adaptability, Source, Cost, Relative Advantage***

***Inner Setting – Relational Connections, Compatibility, Mission Alignment, Relative Priority, Tension for Change, Available Resources, Access to Knowledge and Information***

***Process – Reflecting and Evaluating, Engaging***

***Outer Setting – External Pressure, Policies and Laws, Local Conditions***

- Description of ambulatory pharmacy program(s)
  - Who is involved in the program(s)
  - What are key components of the program(s)
  - How is the program(s) integrated into routine clinical care
  - Monitoring of program(s)
  - Funding/financing of the program(s)
- Description of implementation of the program
  - Motivations for ambulatory pharmacy program(s)
  - Program champion
  - Who else was involved in setting up the program
  - How program has changed over time
- Changes to care delivery associated with ambulatory pharmacy program(s)
  - Support/resistance from providers
  - Training or other education to establish program
  - “Hard wiring” into routine clinical care (i.e., creation of referral pathway, order set, etc.)
    - Institutional versus individual “hard wiring”
  - Success of integration into care
- Factors that facilitated the implementation, expansion, or dissemination of the program(s)
  - Leadership support
  - Champion support
  - Financial support
  - Integration into clinical workflow
  - Existing infrastructure or experience
  - Policy/regulatory requirements
- Barriers to implementing, expanding, or disseminating the program(s)
  - Staff time
  - Costs
  - Patient need/uptake
    - Patient response
  - Knowledge of program(s)
- Adaptations made to fit the ambulatory pharmacy program(s) to your local context

***Innovation Trialability, Cost, Complexity***

- Sustainability/expansion of the program(s) going forward
  - Financing
  - Staff time
  - Leadership support
  - Policy/regulatory requirements
